# Clinical and Pre-Clinical Evidence of Carbonic Anhydrase IX in Pancreatic Cancer and Its High Expression in Pre-Cancerous Lesions

**DOI:** 10.3390/cancers12082005

**Published:** 2020-07-22

**Authors:** Sabina Strapcova, Martina Takacova, Lucia Csaderova, Paola Martinelli, Lubomira Lukacikova, Viliam Gal, Juraj Kopacek, Eliska Svastova

**Affiliations:** 1 Department of Tumor Biology, Institute of Virology, Biomedical Research Center, Slovak Academy of Sciences, Dubravska cesta 9, 84505 Bratislava, Slovakia; sabina.strapcova@gmail.com (S.S.); martina.takacova@savba.sk (M.T.); lucia.csaderova@savba.sk (L.C.); lubomira.lukacikova@savba.sk (L.L.); virukopa@savba.sk (J.K.); 2 Institute of Cancer Research, Clinic of Internal Medicine I, Medical University of Vienna, 1090 Vienna, Austria; paola.martinelli@boehringer-ingelheim.com; 3 Cancer Cell Signaling, Boehringer-Ingelheim RCV Vienna, A-1121 Vienna, Austria; 4 Alpha Medical Pathology, Ruzinovska 6, 82606 Bratislava, Slovakia; viliam.gal@gmail.com

**Keywords:** carbonic anhydrase IX, pancreatic cancer, intraductal papillary mucinous neoplasia, acidic microenvironment, hypoxia, correlation analysis, immunohistochemistry

## Abstract

Hypoxia is a common phenomenon that occurs in most solid tumors. Regardless of tumor origin, the evolution of a hypoxia-adapted phenotype is critical for invasive cancer development. Pancreatic ductal adenocarcinoma is also characterized by hypoxia, desmoplasia, and the presence of necrosis, predicting poor outcome. Carbonic anhydrase IX (CAIX) is one of the most strict hypoxia regulated genes which plays a key role in the adaptation of cancer cells to hypoxia and acidosis. Here, we summarize clinical data showing that CAIX expression is associated with tumor necrosis, vascularization, expression of Frizzled-1, mucins, or proteins involved in glycolysis, and inevitably, poor prognosis of pancreatic cancer patients. We also describe the transcriptional regulation of CAIX in relation to signaling pathways activated in pancreatic cancers. A large part deals with the preclinical evidence supporting the relevance of CAIX in processes leading to the aggressive behavior of pancreatic tumors. Furthermore, we focus on CAIX occurrence in pre-cancerous lesions, and for the first time, we describe CAIX expression within intraductal papillary mucinous neoplasia. Our review concludes with a detailed account of clinical trials implicating that treatment consisting of conventionally used therapies combined with CAIX targeting could result in an improved anti-cancer response in pancreatic cancer patients.

## 1. Introduction

**Figure 1:**
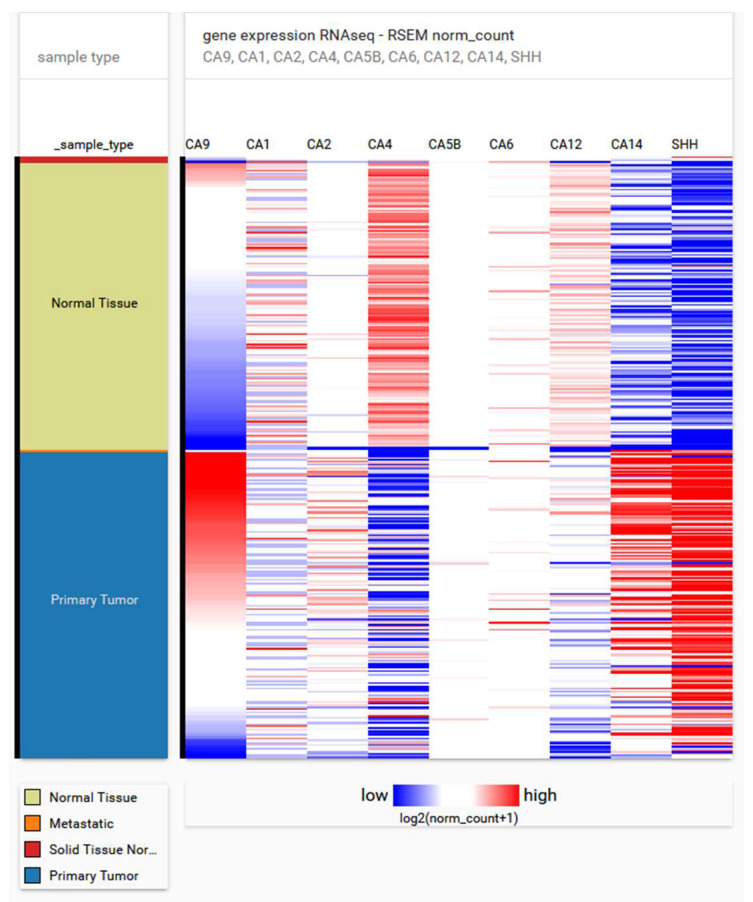
The expression profile of selected carbonic anhydrase isoforms and sonic hedgehog (activated in pancreatic cancer) in 179 samples of pancreatic adenocarcinoma and 171 normal pancreatic tissue samples extracted from Xena (using the cancer genome atlas (TCGA) target GTEx datasets) [15] (for more information see Supplementary Material). Expression of CA9 increases in pancreatic ductal adenocarcinoma samples along with Sonic Hedgehog (SHH) level. Analysis of CA9 promoter revealed possible regulation through Sonic Hedgehog (SHH) pathway (see Figure 2). According to the carbonic anhydrase nomenclature, human CA isoenzymes are written in capital Roman letters and numerals, while their genes are written in Italic letters and Arabic numerals.

To date, several members of the carbonic anhydrase (CA) family namely CAI, CAII, CAIV, CAVB, CAVI, CAIX, and CAXII, have been identified in the human pancreas. Within the organ, CAs localize in alpha cells of Langerhans’s islets (cytosolic CAI [1]), beta cells of the pancreas (mitochondrial CAV [2]), acinar cells (secreted CAVI [3], transmembrane CAXII [4]), and pancreatic ducts (cytosolic CAII [1,5], transmembrane CAIX [6,7], transmembrane CAXII [4]). CAXIV has no expression in healthy pancreas, but it has been found in pancreatic tumors [8] (Figure 1).

As with most carbonic anhydrases, CAIX catalyzes a simple physiological reaction, the reversible hydration of carbon dioxide to bicarbonate ions and protons. Hence, this exofacial CA located at the cell membrane is a crucial component of the pH-regulating machinery in tumors [9,10,11]. To prevent the build-up of acidic metabolites inside cancer cells and to maintain a permissive intracellular pH for cell proliferation and survival, CAIX closely cooperates with the bicarbonate transporters NBCe1 and NBCn1, catalyzing HCO^3−^ influx [12,13]. This functional interaction increases the efficiency of ion transport through a plasma membrane, generation of pH gradient, and neutralization of intracellular space. Together with CAIX, monocarboxylate transporters (MCTs) are also thought to participate in the process of extracellular acidification by exporting lactate and ions [14].

The reversed intracellular (pHi) and extracellular pH (pHe) gradient generated by the cooperation of CAIX with other proteins enhances cell migration, during which CAIX relocalizes to lammelipodia [12]. Invadopodial distribution of CAIX promoting the proteolytic activity of matrix metalloproteasis within invasion through the extracellular matrix and quail chorioallantoic membranes, as well as the ability of anti-CAIX antibodies to cease this process, was shown by Debreova et al. (2019) [16] (the role in invadopodia was also described in [17]). Furthermore, CAIX de-stabilizes cell-cell adhesion through competitive binding with β-catenin, disabling the formation of a complex between β-catenin and E-cadherin necessary for adherent junction maintenance. The disruption of this complex facilitates tumor cell dissemination and further cancer progression [16,18]. Interestingly, CAIX is also cleaved from the cell membrane by a disintegrin and metalloproteinase 17 (ADAM17). Its ectodomain serves as a potential blood biomarker of some cancer types with developed hypoxia [19,20].

Considering its unique transcriptional regulation and expression, enzymatic activity, as well as appertaining functions [21], CAIX possesses a special position among all human CAs and is a crucial player implicated in cancer pathogenesis due to its catalytic and non-catalytic mechanisms. While enzymatic activity is mediated by the protein’s catalytic CA domain oriented towards the extracellular space, the unique presence of a proteoglycan-like (PG) domain enables non-catalytic functions characteristic of CAIX. The PG domain is negatively charged and easily dissociates from complexes at a slightly acidic pH [22], facilitating the detachment of primary tumor cells, and eventually leading to the formation of metastases. Through its PG domain, CAIX facilitates adhesion to collagen. During initial adhesion and spreading, it colocalizes with paxillin in focal contacts [23]. Moreover, the PG domain displays the capability to bind mucins, abundantly overexpressed in pancreatic tumors.

The expression pattern of CAIX clearly differs from the rest of its CA family members: CAIX can be found in only a few normal tissues, but its ectopic expression is strongly connected with many cancer types, primarily due to the presence of hypoxia. The unique localization of a hypoxia response element ensures that CA9 belongs to the group of genes with the strongest hypoxia response executed via hypoxia-inducible factor 1 (HIF-1) [24]. In pancreatic cancer tissue, CAIX expression is higher compared to the healthy organ and precursor lesions [25,26,27] and positively correlates with tumor size and staging [26]. Particularly strong CAIX staining can be observed in areas of pancreatitis adjacent to the invading tumor [25]. High CAIX expression serves as an indicator of worse prognosis [26], but particularly poor prognosis was shown for pancreatic ductal adenocarcinoma (PDAC) patients with high levels of both CAIX and MCT4 in the stroma [28]. In patient-derived xenograft models of pancreatic cancer, CAIX was shown to be a key modulator of cancer initiation and tumor growth [29].

At time of diagnosis, 11% of pancreatic cancers are confined to their primary site and 52% have already metastasized [30]. While the relative 5-year survival for localized pancreatic cancer is 39.4%, for cases staged as distant at diagnosis it is only 2.9% [30]. PDAC is the most common neoplasm of the organ, accounting for 85% of all histologically-confirmed pancreatic cancer cases [31]. This particular subtype of pancreatic cancer has a very poor prognosis—24% of patients live 1 year after diagnosis and only 9% are still alive after 5 years [32].

Even though the 5-year relative survival rate for all pancreatic cancer cases has quadrupled in the last 45 years, it currently remains at a dismal rate of 10% [30], with life expectancy at the time of diagnosis an alarming 4.6 months [33]. With the lowest survival rate of all types of cancer in Europe [34], pancreatic cancer is responsible for more than 95,000 deaths in the EU every year [35]. By the year 2040, the number of cases of pancreatic cancer worldwide is predicted to rise by almost 80% [36].

An established model of pancreatic carcinogenesis resulting in the development of PDAC includes three morphologically-distinctive precursor lesions: pancreatic intraepithelial neoplasia (PanIN), intraductal papillary mucinous neoplasm (IPMN), and mucinous cystic neoplasm (MCN) [37]. PanINs arise from pancreatic ducts and are characterized by mucin-containing cuboidal to columnar cells and are graded (PanIN I–III) on the basis of histological and cytological criteria; high-grade PanINs ultimately transform into PDAC [38]. While roughly 40% of PanIN I lesions harbor a K-ras mutation [39,40], inactivating mutations of CDKN2A, TP53, and SMAD4 have a rising incidence with higher PanIN grades [38]. IPMNs (mucin-producing non-invasive neoplasms) usually have a papillary architecture and can arise from the main pancreatic duct or branch ducts. While at cellular level they resemble PanINs, they have the ability to grow into larger cystic structures [41]. The excellent prognosis of IPMNs critically worsens once they progress to invasive carcinoma (40% of IPMN) [42].

One of the reasons accounting for the lethality of pancreatic cancer is its intrinsic or acquired therapy resistance, whose the underlying mechanisms have already been reviewed in [43,44,45]. The major factors contributing to decreased therapeutic efficacy in this malignancy include hypoxia [46] and metabolic reprogramming—both indisputably linked to CAIX expression [47]. Intratumoral hypoxia not only hinders radiotherapy, but also chemotherapy and immunotherapy [48,49,50,51]. CAIX also aids microenvironmental acidification and lactate accumulation, which, in turn, leads to the suppression of the T-cell adaptive immune response and to an overall immune destruction escape [52]. Alterations in pH values within the tumor mass (namely the reversed pH gradient across cell membranes), which are maintained by CAIX in cooperation with other proton pumps and proton transporters, correlate with multi-drug resistance [53].

## 2. Signaling Pathways Activated in Pancreatic Tumors and Their Relationship to Transcriptional Regulation of CA9

The expression of CA9 is critically dependent on the transcription factors (TFs) and their binding sites, which are located within the promoter sequence localized approximately 170 nucleotides upstream from the transcription start site (TSS). A unique localization of a hypoxia response element (HRE) in position −10/−3 from TSS ensures that CA9 belongs to the group of genes with the strongest hypoxia response, which is executed via hypoxia-inducible factor 1 (HIF-1) [24]. HIF-1 is a protein complex composed of two constitutively-expressed α and β subunits, from which the former one is oxygen sensitive. Under hypoxic conditions, β subunit dimerizes with α subunit, leading to the formation of the heterodimeric TF HIF-1, which recognizes and binds the HRE regions of its target genes and upregulates their expression.

Therefore, HRE sequence is the most critical regulatory element within the CA9 promoter, while HIF-1 is a central regulator of CA9 transcription. Several studies have reported that hypoxia is evident in PDAC, in which the expression of HIF-1α and CAIX have been detected in 60–70% and 78% of cases, respectively [25,54,55]. Moreover, Hiraoka et al. confirmed CAIX expression in and around the necrotic lesions and suggested that histological necrosis could be a simple and reproducible predictor of postoperative outcome in PDAC patients [56]. In contrast to previous studies, no correlation between HIF-1α and CAIX expression levels were found by Leppänen and colleagues and, moreover, weak HIF-1α expression indicated poor prognosis for 69 patients with resectable PDAC [57]. A possible explanation for such discrepant findings about the prognostic role of HIF-1α could lie in the methodology of immunohistochemistry, as well as in the primary antibodies used.

**Figure 2:**
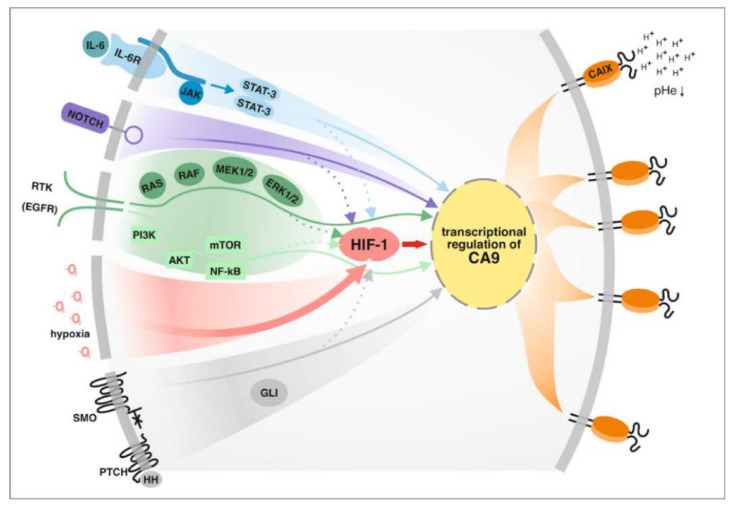
Schematic presentation of signaling pathways activated in pancreatic cancer and their effect on CA9 expression.

Besides HIF-1 and its binding within the HRE sequence, the core CA9 promoter contains additional binding elements for several TFs whose binding has already been confirmed (e.g., SP1, AP1 and AP2, ETS-1), or is still waiting for identification [58,59,60]. In addition to hypoxia, CA9 expression is elevated by high cell density and is modulated by the PI3K (phosphatidyl inositol 3-kinase) and MAPK (mitogen-activated protein kinase) pathways [61,62] (Figure 2).

Although several studies have been interested in the immunohistochemical staining of CAIX within pancreatic tumors, very little is known about the transcriptional regulation of CA9. So far, the major concern has been to study and affect hypoxia and HIF-1 presented in pancreatic tumor cells. For that reason, CA9 mRNA as well as CAIX protein expression as an exclusive HIF-1 target were analyzed. This example represents a specific small-molecule inhibitor—APX3330 (also called E3330) —of AP endonuclease1/Redox effector factor 1 (APE1/Ref-1), which was confirmed to reduce oxidized TFs and consequently to target the downstream signaling pathways. Fishel and colleagues showed that blocking APE1/Ref-1 redox signaling via E3330 leads to a decrease in TF activity of NF-κB, AP-1, and HIF-1α in vitro [63]. Dose-dependent inhibition of the HIF-1 signaling pathway after the treatment of Panc-1 and PaCa-2 cells with E3330 was confirmed on the CA9 mRNA level using real-time quantitative reverse transcription PCR (qRT-PCR). Moreover, a combination treatment with APE1/Ref-1 inhibitor E3330 and CAIX inhibitor SLC-0111 (Clinical Trial NCT02215850) was shown to reduce proliferation and size of three-dimensional spheroids derived from PDAC patients [64]. In line with these findings, the application of novel analogues of both APE1/Ref-1 and CAIX inhibitors (namely APX2009, APX2014, and FC12-531A) with improved potency significantly affected pancreatic cancer cell survival [65].

Key players contributing to pancreatic cancer development and progression are inflammatory cytokines produced by tumor and inflammatory cells, as well as cancer associated fibroblasts. Paracrine signaling plays a critical role in the whole secretome regulating desmoplasia and pancreatic cancer behavior. Soluble mediators like IL-6, IL-8, IL-1β, TGF-β, TNF-α, VEGF, SHH, etc. have been implicated in pancreatic cancer carcinogenesis [66]. A multifunctional cytokine interleukin (IL)-6 which activates upon binding to its receptor, a Janus kinase (JAK) family of tyrosine kinases and consequently stimulates multiple signaling pathways, e.gi. MAPK, PI3K, STAT [67], was shown to induce also CA9 expression. Using the model of human mammospheres, Sansone and collaborators revealed IL-6-induced Notch-3-dependent up-regulation of CAIX promoting the malignancy in breast cancer stem cells [68]. Moreover, they demonstrated that an interplay between p66Shc and Notch-3 regulates hypoxia response (via upregulation of CAIX) and the control of stem cell survival (via upregulation of the Notch ligand Jagged-1) [69]. In line with previous data, the relevance of co-operation between the intracellular domain of Notch-3 (NICD) and HIF-1α for CA9 expression (at the level of mRNA, protein, and reporter) was described in breast cancer cells [70]. Enhanced cancer cell growth rates and invasiveness in an IL-6-dependent manner (through activation of Notch-3, Jagged-1, and CAIX via STAT-3) was reported by Studebaker [71]. In accordance with the results gained for breast cancer, a positive correlation between phosphorylation of STAT-3, Her-2 status, CAIX expression, and prognosis was observed in a large cohort of esophageal carcinomas [72]. Given the reported involvement of IL-6 in upregulation of CAIX expression in multiple previously-mentioned cancer models, it seems plausible that IL-6 signaling could also be implicated in the transcriptional regulation of CA9 in precursor lesions as well as in primary pancreatic tumors.

In addition to IL-6, STAT-3 can be activated by another receptor and non-receptor tyrosine kinases, such as epidermal growth factor receptor (EGFR), whose overexpression in pancreatic tumorigenesis has been implicated in neoplastic precursors and additionally, after tumor initiation, in the maintenance of MAPK/ERK activity [73,74]. Similarly to MAPK/ERK, PI3K/AKT represents another downstream signaling pathway which mediates the biological response of the EGFR. Both above-mentioned signaling pathways were previously reported to be involved in the transcriptional regulation of CA9 under hypoxia and high cell density [61,62]. In line with this, McDonald and colleagues showed that K-ras knockout pancreatic cancer cells reduced the protein level of HIF-1α, CAIX, and glycolysis in response to hypoxia [75]. Interestingly, knocking out K-ras as well as treatment with the mitogen-activated protein kinase (MEK) inhibitor trametinib lowered HIF-1α, CAIX, and phosphorylation of ERK1/2 even in normoxia. Hypoxia is a major pathophysiologic stimulus for upregulation of CA9 in solid tumors. However, HIF-1α expression can also be stabilized/induced through other effectors and pathways, including PI3Kinase and mammalian target of rapamycin (mTOR), mutations in the tricarboxylic acid proteins such as fumarate hydratase, and gemcitabine [75].

It is well known that hypoxia plays a crucial role in cancer epithelial-to-mesenchymal transition (EMT) and invasion. A possible explanation of how hypoxia may contribute to these events lies in the activation of the hedgehog (HH) signaling pathway. While the HH pathway is normally quiescent in adult pancreatic tissue, it has been shown to activate in pancreatic cancer cells where it promotes stromal hyperplasia and the production of the extracellular matrix (as reviewed in [76,77]). Bailey and colleagues revealed that Sonic Hedgehog (SHH) expression promotes a desmoplastic reaction in pancreatic cancer [78]. Interestingly, Spivak-Kroizman and collaborators revealed that elevated secretion of SHH ligand by cancer cells (mediated via HIF-1α) could be responsible for the prevalence of desmoplasia in this type of cancer. In line with this, elevated levels of HIF-1α and HH were found in pancreatic tumors and identified as markers of decreased patient survival [79].

SHH and downstream components of the HH pathway have been shown to be upregulated in precursor lesions and primary pancreatic tumors, but not in the normal pancreas [80]. Moreover, analysis of the HH pathway in IPMN tissue and xenografts showed that activation of the hedgehog signaling pathway represents an important step in the development of IPMN [81,82]. Interestingly, the nuclear expression of GLI1 was described to be elevated upon exposure to hypoxia, suggesting that GLI1 transcription factor could directly mediate hypoxia-induced EMT and invasion [83]. Several studies have revealed that a crosstalk between hypoxia and HH pathways within pancreatic cancer cells is operated in a ligand-independent manner and that the nuclear accumulation of GLI1 could be triggered via other factors, such as TGF-β, K-ras, and receptor tyrosine kinase (RTK) (reviewed in [84]). Such a non-canonical mechanism of GLI activation could contribute to the development of several types of cancer. PDAC, characterized by a high incidence of activating K-ras mutations, represents an exclusive example of non-canonical induction of the HH signaling pathway.

Hypoxia has been shown to increase HH pathway activation through the upregulation of SHH, Smoothened (SMO), and GLI1 transcription in a ligand-independent manner, leading to enhanced invasiveness of pancreatic cancer [85]. In addition, the expression of MMP9, which contributes to the invasiveness of pancreatic cancer cells, was revealed to be affected via the HH signaling pathway alone, and in combination with hypoxia [85,86]. GLI1 is a transcriptional activator of target genes, and is itself a transcriptional target of the HH pathway. As a member of the GLI-Kruppel family of zinc-finger containing TFs, GLI1 binds specifically to a 9-base pair DNA sequence 5′-GACCACCCA-3′ within the target gene promoters [87]. Interestingly, only the two cytosine pairs flanking the central adenine within the consensus site are critical for GLI binding, whereas the other positions can tolerate a certain degree of flexibility [88].

**Figure 3:**
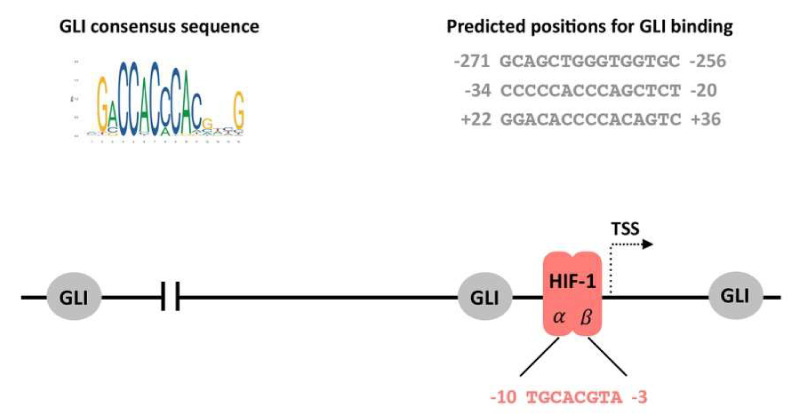
GLI consensus sequence (left side) and prediction of GLI binding sites (right side) within the CA9 promoter were acquired using JASPAR software. The position of three predicted GLI binding sites is outlined in relation to the hypoxia-responsive element (HRE; the sequence recognized by HIF-1 transcription factor) located −10/−3 upstream from the transcription start site (TSS).

Considering all previously-mentioned indications, we performed a comprehensive in silico analysis of the CA9 promoter sequence using JASPAR [89] and MatInspector [90,91] (Supplementary Material). Through the JASPAR database, we revealed three putative GLI binding sites within the CA9 regulatory sequence (relative profile threshold score was 80%, two of them were upstream and one was located downstream from the TSS; Figure 3). However, using a MatInspector analysis of the same CA9 promoter sequence, we were able to reveal only one putative GLI binding site at position −270/−256 upstream from the TSS, having the matrix similarity 0.938 and the highest core similarity 1. Such discrepancies could be explained by the sensitivity and specificity, as well as the matrix description, which do not reflect binding sites in their genomic context. Although all these binding sites could possibly be recognized by GLI transcription factor, it is important to mention that their functionality and relevance for CA9 transcription has to be verified experimentally.

Interestingly, some indications supporting the role of the HH signaling pathway in upregulation of CA9 expression can be found in several clinical studies. Onishi and colleagues confirmed, using triple staining fluorescence immunohistochemistry of surgically-resected pancreatic cancer tissues, that GLI1 and SMO proteins are partially co-expressed with CAIX, a marker of hypoxia [85]. Furthermore, Couvelard and colleagues observed 48% stromal cell positivity for CAIX in a cohort of 50 patients, as well as CAIX-positive fibroblasts in PDAC-possessing fibrotic areas. Since stromal HIF-1α expression in fibroblasts was detected in a very low proportion of PDAC patients, and positive correlation was revealed between stromal CAIX positivity and CAIX+ cancer cell areas (predominantly upregulated via HIF-1) [25], we can expect an additional regulatory mechanism (e.g., paracrine stimulation of CAIX expression in stromal cells), which is mediated via hypoxia-induced HH signaling from adjacent tumor cells. Taken together, all these data suggest that the expression of CAIX in precursor lesions, as well as in primary pancreatic tumors, could be at least partially affected by HH signaling pathway activity, either alone or in collaboration with hypoxia and HIF-1.

In conclusion, hypoxia and the transcription factor HIF-1 still remain the major transcriptional regulators of CA9 expression. However, the absence of correlation between HIF-1 and CAIX supports the hypothesis that rather than being regulated by hypoxia, CAIX expression could be induced by other signaling pathways and transcription factors. Therefore, the activity of IL-6/STAT-3, EGFR, MAPK, PI3K, as well as HH/GLI, which were all described as essential for pancreatic carcinogenesis, could be relevant for CA9 transcription and should be taken into consideration, especially in situations with no oxygen restrictions. Schematic presentation of signaling pathways relevant in pancreatic cancer and their effect on CA9 expression is illustrated in Figure 2.

## 3. Inflammation as an Inherent Feature of Pancreatic Cancer and Its Impact on CAIX Expression

The risk factors for pancreatic cancer development include genetic, environmental, and medical factors [92]. Pancreatitis, characterized by premature activation of digestive proenzymes and a subsequent autodigestion of the organ [93], remains the most significant risk factor, with the chance of pancreatic cancer development drastically increasing in patients with longer-standing chronic pancreatitis [92]. Even though only a minority of PDAC patients have a clinical history of pancreatitis [94,95], pancreatitis-induced stress was shown to cause acinar to ductal metaplasia [96,97,98,99] which can lead to the formation of PanIN lesions [100].

Damage-associated molecular patterns (DAMPs) are released upon stress, e.g., cellular injury, inflammation, or tissue damage [101] to stimulate toll-like receptors (TLRs), a subgroup of pattern recognition receptors (PRRs). TLRs enable cancer cells to undergo the switch from oxidative phosphorylation to glycolysis [102] and drive pancreatic stromal inflammation [103]. Toll-like receptor expression depends on PanIN grade [104], increases as cancer progresses [105], and is associated with metastases [106]. TLRs activate the NF-κB [101,107,108] and MAPK signaling pathways [108]. The subsequent cyclooxygenase (COX)-2 production [107] is vital for malignant transformation of normal cells, reduced apoptosis [109], invasion, and angiogenesis [110]. In pancreatic cancer, TLR activation also aids cancer cell proliferation and reduces chemosensitivity [105]. Through PGE2 synthesis and subsequent activation of the MAPK signaling pathway, COX-2 might upregulate CAIX expression and promote an aggressive cancer phenotype, similar to colorectal cancer cells where the expression of COX-2 and CAIX has been shown to correlate with each other and increase with tumor stage [111].

Inflammatory process initiation is further mediated by pro-inflammatory cytokines and chemokines, which can be released by acinar cells, neutrophils, monocytes, and eventually even by cancer cells, as well as other cells residing in the tumor microenvironment (TME). Inflammatory molecules such as IL-1, IL-6, TNF-α, and TGF-β [112,113,114] can activate pancreatic stellate cells which serve as a principal source of fibrosis [115]. The emerging fibrotic foci correlate with the presence of CAIX, localized in stromal fibroblast and in roughly 80% of tumors [25].

Crosstalk between TLR expression (i.e., innate immunity response, hypoxia, and CAIX expression) already exists in early pancreatic carcinogenesis [104]. The transcription factor HIF-1α activates expression of several TLRs under hypoxic conditions [116,117,118]. The expressions of some TLRs and HIF-1α correlate [104,119] and prominent expression of HIF-1α, TLRs, and CAIX can be found as early as in PanINs [104]. Conversely, TLR3 was shown to induce the Warburg effect and aid tumor cells in adapting to the hypoxic milieu [102], whereas HIF-1α and TLR4 may synergistically promote development of pancreatic cancer [119].

While the most potent regulator of CAIX expression is hypoxia, factors that can activate the transcription factor HIF-1 in normoxia include reactive oxygen species, succinate, and lactate [120]. In densely-grown cell cultures, minimal HIF-1 levels are required to initiate CAIX expression, as long as the transcription factor SP1 is present [58,62]. Similarly, the pro-inflammatory cytokine IL-6 is able to induce CAIX expression even in the absence of hypoxia [68]. CAIX is thought to be involved in promoting survival and enhancing the invasive behavior of cancer cells via the IL-6/Notch-3/CAIX axis [68].

Vast evidence suggests the involvement of CAIX in pancreatic cancer pathogenesis. Its localization at fibrotic sites and areas of pancreatitis, the ability to be induced in both a hypoxia-dependent and independent manner, the existence of crosstalk between CAIX and inflammatory molecules, as well as its correlation with necrosis, tumor grade, and poor prognosis [25] places CAIX in the center of pathological events leading from malignant transformation to pancreatic cancer progression and metastasis.

## 4. Clinical Significance of CAIX in Pancreatic Cancer

Expression of CAIX in non-neoplastic areas of the pancreas is very weak, while its abundance and intensity increase in pancreatitis regions close to invading tumors [4,25,121]. The rise in incidence and intensity of CAIX staining continues toward pancreatic ductal adenocarcinomas. The presence of CAIX in PDAC patients has been demonstrated in many papers, with its localization also detected in tumor and stromal cells. In a cohort of 50 patients with PDAC, Couvelard et al. showed CAIX positivity in 78% of cases [25]. Importantly, expression of CAIX correlated with fibrotic focus, which histologically reflects intratumoral hypoxia, replacing necrosis. Both the expression of CAIX and the presence of fibrotic focus were associated with lower survival. Interestingly, despite HIF-1α positivity in 66% of patients, the authors claim no association between HIF-1α and its downstream targets CA9 and VEGF, or clinicopathological variables. Similarly, immunohistochemical analysis of PDAC patient tissues confirmed the association of CAIX positive areas with necrosis, as a consequence of hypoxic occurrence. When micro- and macro-necrosis were taken into account, more than 60% of samples displayed necrotic areas. CAIX expression and necrosis were significantly associated with shorter disease-free survival and disease-specific survival, and were independently worse prognostic factors in a cohort of 348 PDAC patients [56].

Furthermore, CAIX expression was also associated with VEGF and with an increased microvessel density (MVD). Studies evaluating the significance of vasculature as a prognostic factor of metastasizing emphasize that a combination of α-SMA and CD34 (MVD marker), indicating microvessel integrity, is more critical for cancer cell invasion and their dissemination through blood vessels. In patients with high MVD coupled with high microvessel integrity (high α-SMA), the metastatic probability could be low [122]. A total of 179 pancreatic cancer patients were divided into 4 subgroups of different microvessel integrity (MVI). The largest HIF-1α and CAIX density was detected in two subgroups with the worst overall survival (OS) and low microvessel integrity (low α-SMA, high MVD and low α-SMA, low MVD), where only 30% of patients survived the 7-month time point. Conversely, the lowest intensity of HIF-1α and CAIX was in the subgroup with high MVI (high α-SMA, high MVD), with more than 90% of patients still alive at the 18-month time point. Similar results were confirmed in hepatocellular carcinoma, which are highly vascularized in contrast to pancreatic cancer, emphasizing the importance of MVI instead of CD34-based vascularization in tumor recurrence and metastasis [122].

**Figure 4:**
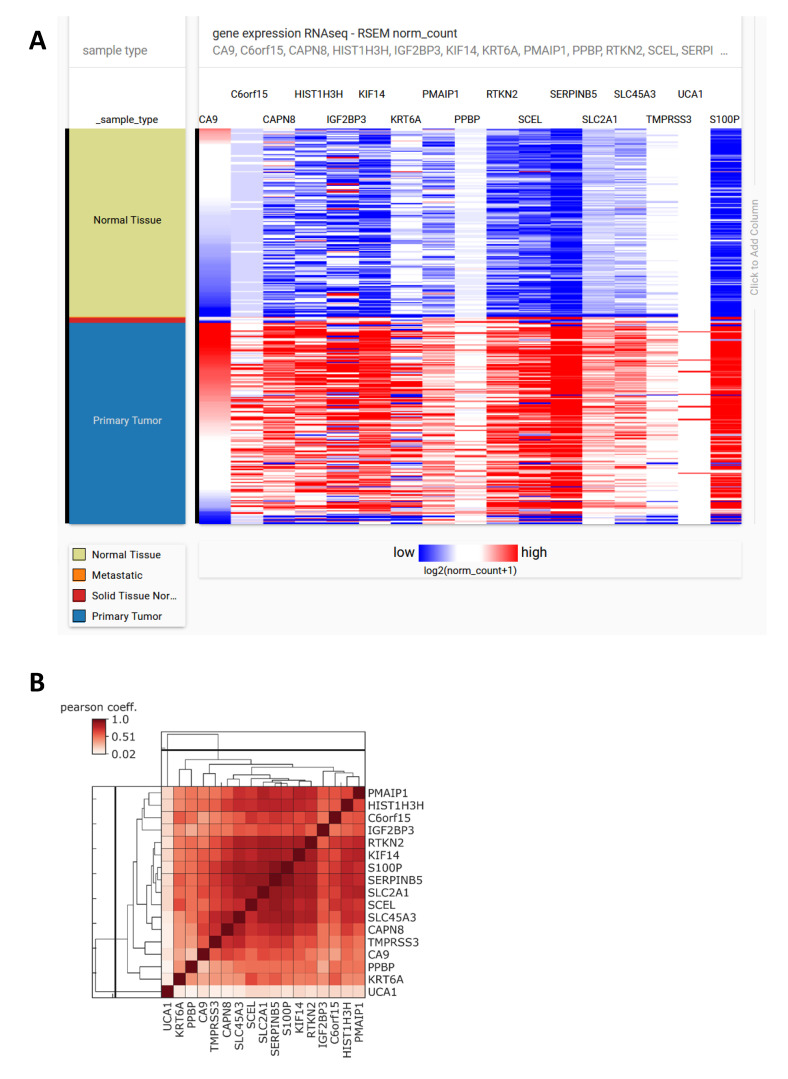
(**A**) The expression profile of CA9, S100P, and prognostic 15 gene signature panel for Early Stage Pancreatic Ductal Adenocarcinoma in 179 samples of pancreatic adenocarcinoma and 171 of normal pancreatic tissue extracted from Xena (TCGA target GTEx). (**B**) Pearson correlation coefficient diagram of analyzed genes. CA9 is positively correlated with the 15 gene signature panel as well as S100P (Supplementary Material).

A prognostic gene signature was derived for patients suffering from early-stage PDAC, using overall survival as a primary endpoint [123]. The set is comprised of 15 genes whose higher expression was associated with poor overall survival. Six genes (IGF2BP3, KIF14, PPBP, SERPINB5, SLC2A1, TMPRSS3) out of 15 were linked with differing outcomes in other independent studies, indicating the possibility of using the signature as a prognostic tool specific to PDAC. Our in silico analysis of 179 PDAC samples and 171 normal pancreas samples derived from the cancer genome atlas (TCGA) and GTEx datasets showed upregulation of all analyzed genes as well as CA9 in tumor samples compared to healthy tissues (Figure 4A). We detected a positive correlation of CA9 expression with all 15 genes of the panel and with S100P, which is also implicated in PDAC tumorigenesis (Figure 4B). A correlation coefficient of 0.5 and higher was found between CA9 and more than 60% of the analyzed genes.

Research addressing the expression of arginase II (ARG2) in pancreatic cancer and its role in immune suppression revealed that the majority of ARG2 is present in cancer-associated fibroblasts (CAFs) in hypoxic state [124]. ARG2-expressing CAFs were located around necrosis with CAIX, GLUT1, and HIF-1α positivity. Interestingly, areas of ARG2 and thus also CAIX-expressing CAFs significantly correlated with an immunosuppressive microenvironment identified by higher infiltration of CD68^+^ macrophages, CD66b^+^ neutrophils, and lower infiltration of CD4^+^ T cells and CD8^+^ T cells. Increased ARG2 expression and a diminished number of tumor-infiltrating lymphocytes was also reported in prostate cancer [125]. It is probable that in reported cases of PDAC patients, the precise combination of increased ARG2 with a hypoxic TME, characterized by CAIX expression and known for its immunosuppressive properties, contributes to the inhibition of an anti-tumor immune response. This clinical study implies that CAIX protein through its impact on extracellular acidosis contributes to the inhibition of immune cell infiltration.

Epigenetic changes, which are strongly affected by hypoxia, are an important feature of cancer development and progression [126]. Hypoxia upregulates the expression of different DNA methylation and demethylation enzymes. Expression of CAIX as a hypoxia marker was also investigated in relation to the methylation status of mucins promoter regions, which are aberrantly produced and play a crucial role in pancreatic cancer carcinogenesis. Expression of MUC1, MUC4, and MUC5AC increases progressively with the advancement of pancreatic cancer, and is associated with poor survival [127]. Importantly, MUC1 and MUC4 modulate chemoresistance of pancreatic cancer cell lines in vitro. Their downregulation enhances sensitivity to gemcitabine, the leading chemotherapy in pancreatic cancers [128].

Yokoyama et al. revealed high correlation between the expression of CAIX, MUC1, and MUC4 in pancreatic cancer patients [129]. They showed that hypomethylation of MUC1 and MUC4 promoters correlates with mRNA and IHC positivity of both mucins in pancreatic cancer tissues, and with a high CA9 level. Analysis of overall survival showed that patients with hypomethylation of MUC1 and MUC4 display worse prognosis compared to those with hypermethylation. Furthermore, the correlation between CA9 expression as a hypoxic environment marker and DNA demethylation enzymes TET1 and TET2, and DNA methyltransferase DNMT3a, was confirmed in pancreatic neoplastic tissues. TET1, TET2, and DNMT3a also correlated with the hypomethylation status of MUC1 and MUC4, and with the development of distant metastasis. Thus, CAIX as a marker of hypoxic TME indicates tumor milieu, where epigenetic changes are one of the mechanisms regulating pancreatic cancer progression. Association of methylation status with expression of different mucins was also shown in breast, lung, pancreas, and colon cancer cell lines [130]. Global reduction of DNA methylation in hypoxic regions of tumors was clearly demonstrated in mouse xenografts of colorectal and melanoma cancers [131]. The authors found an inverse correlation between DNA methylation in cancer cells and their distance to perfused vessels. Contrarily, other studies reported that hypoxia promotes DNA hypermethylation in a panel of human cancers [132]. Importantly, Thienpont et al. reported that tumor suppressor genes and oncogenes are differently affected by hypoxia-induced DNA methylation, with strikingly more methylated promoters of tumor suppressor genes in hypoxic tumor areas [133].

In addition to hypoxia, hedgehog and Wnt signaling are improperly upregulated in pancreatic cancer, even in pre-cancerous lesions. Moreover, the Wnt cascade is also regulated by the hypoxia and hedgehog pathways. Canonical Wnt signaling is triggered by the complex formation of soluble Wnt ligands with their Frizzled receptors and LRP5/LRP6 co-receptors, resulting in β-catenin/TCF-LEF transcriptional activity. Activation of Wnt target genes facilitates proliferation, invasion, and EMT. Overexpression of Frizzled receptors (FZD1, 2, 7, 9) and their ligands (WNT2, 3, 4, 5A, 5B, etc.) was detected in pancreatic cancer patients compared to those with a normal pancreas. Importantly, downstream target genes of the Wnt pathway were also strongly upregulated (cyclinD1, fibronectin, MMP7, COX-2, uPAR, etc.), indicating activated Wnt signaling in PDAC [134]. Yang et al. showed that 56.6% and 54.7% of PDAC patients (*n* = 106) were positive for CAIX or FZD1, respectively [27]. The expression of FZD1 positively correlated with CAIX, whilst out of all FZD1 positive cases, 79% had positive CAIX expression (as for FZD1 negative samples, 70% were CAIX negative). The authors considered as positive those tissues that contained ≥25% immunohistochemically positive cells (they did not examine staining intensity). Based on this approach, expression of CAIX as well as FZD1 correlated with clinicopathological characteristics of pancreatic ductal adenocarcinoma such as TNM stage, lymph node metastasis, and lymph node invasion. Positive FZD1 and CAIX expression was significantly higher in PDACs than in precursor lesions or a healthy pancreas, and were associated with shorter overall survival.

Although FZD1 was undetected in the study by Zeng et al. [135], FZD2 and Wnt-1 were upregulated concurrently with β-catenin in around 65% of evaluated PDAC tumors. In accordance with the activated Wnt/β-catenin pathway, they also showed a decrease in β-catenin phosphorylation (Ser45/Thr41) responsible for its degradation, with β-catenin/TCF complexes regulating gene transcription only detected in the nuclear extracts of tumors. Another mechanism of Wnt pathway activation described in pancreatic cancer is through the increased level of Disheveled-2 protein, a negative regulator of GSK3β-axin complex, protecting β-catenin from degradation. Both ATDC and IQGAP1 proteins, recently described as overexpressed in PDAC patients, increase stability of DVL-2 with subsequent elevation of free β-catenin pool and its transcriptional activity. In primary pancreatic cancers, the level of ATDC correlates with DVL-2 and increased β-catenin [136]. Hu et al. proved the prognostic value of IQGAP1 expression in PDAC patients [137]. The group with a high IQGAP1 level displayed significantly poorer overall survival rates than the IQGAP1-low group. IQGAP1 directly interacts with DVL-2 and β-catenin, and promotes pancreatic cancer progression and EMT. In a previous work, we showed that CAIX protein co-precipitates with β-catenin, and ectopic expression of CAIX decreases the amount of cytoskeleton-linked E-cadherin, β-catenin, and α-catenin proteins [18]. Thus, CAIX could facilitate pancreatic cancer through the destabilization of cell–cell adhesion and its involvement in Wnt pathway activation.

## 5. CAIX in Pre-Cancerous Lesions of Pancreatic Ductal Adenocarcinoma

Although several papers have reported that a high percentage of normal pancreas tissues express CAIX, it is important to note that this expression is very weak compared to the hyperplastic epithelium, PanIN, or PDAC lesions [25,57]. On the other hand, Kivelä and colleagues observed sporadically-expressed CAIX in the basolateral membranes of normal acinar and ductal epithelial cells, with an increasing and stronger immunoreaction in the hyperplastic epithelium adjacent to tumor tissue [4]. Yang et al. analyzed 55 precursor pancreatic lesions, where among the intraepithelial neoplasia, chronic pancreatitis and adenomas, the positive rate of CAIX was 20%, 10%, and 20% respectively [27]. Expression in normal pancreata was documented in 15.4% of cases. Discrepancies between individual articles mainly arise from different approaches to the evaluation of immunohistochemistry positivity (percentage of positive cells versus complex histoscore calculation), the immunohistochemistry procedure used, and from the use of different primary antibodies. A very detailed investigation of CAIX expression in PanINI to PanINIII was performed by Lepännen et al. [57]. Strong membranous CAIX positive staining was detected in 90% of PanINI, 100% of PanINII, and 94% of PanINIII lesions. The histoscore of CAIX staining increased from PanINI to PanINIII. In contrast, normal acinar and duct cells were CAIX negative or weakly positive, although the positivity was declared in 84% of normal ducts. Importantly, the histoscore in normal pancreatic ducts is almost three times lower than in the most severe lesions (PanINIII). Similarly, nuclear HIF-1α positivity was detected in around 80% of normal and preneoplastic PanIN lesions, but again the histoscore in PanINIII was significantly higher than in normal pancreatic ducts. It is important to note that all these stainings were performed on tissue sections of patients who had already developed pancreatic cancer with clinical manifestations. Paracrine factors produced within the TME combined with inflammatory cytokines might influence adjacent healthy cells compared to pancreata with PanINs but without cancer.

**Figure 5:**
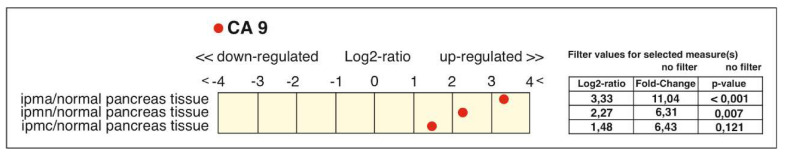
Upregulation of CA9 in intraductal papillary mucinous neoplasm (IPMN) samples compared to normal pancreatic tissue. The analysis was performed in Genevestigator (Nebion AG), a multi-organism microarray data analysis toolbox, using data from experiment No. HS-00705 (GSE19650) comprising 21 samples of intraductal papillary-mucinous adenoma (ipma, *n* = 6), adenocarcinoma (ipmc, *n* = 6), invasive carcinoma (ipmn, *n* = 3), and normal pancreatic duct tissue (*n* = 6). The graph shows a log fold change between various types of mucinous neoplasia tissues and healthy tissue samples; *p* denotes significance of changes.

Besides the previously-mentioned PanINs, other types of pancreatic precursor lesions are intraductal papillary mucinous neoplasm and mucinous cystic neoplasm, which make up 20% of all PDAC precursors. Greater detail about the biology and genetics of IPMNs can be found in several reviews (e.g., [37,138]). Only one recent paper describes intensive immunostaining of CAIX in mucinous cystadenomas (benign cystic tumors with a risk of malignant progression) and weak to strong CAIX positivity in benign microcystic adenomas [27]. Transcriptomic profiling of 12 selectively-microdissected IPMNs observed CA9 as one of the most highly-expressed genes in IPMNs (17-fold increase) among the highest-ranked 100 known genes, as compared to normal pancreatic ducts [139]. Our Genevestigator analysis [140] also showed an increased CA9 level in samples of intraductal papillary mucinous adenoma, adenocarcinoma, and invasive carcinoma from patients with pancreatic cancer compared to normal pancreatic duct tissue (Figure 5). The CA9 upregulation ranges from 6-fold in adenocarcinoma and invasive carcinoma to an 11-fold change in the case of adenoma.

**Figure 6:**
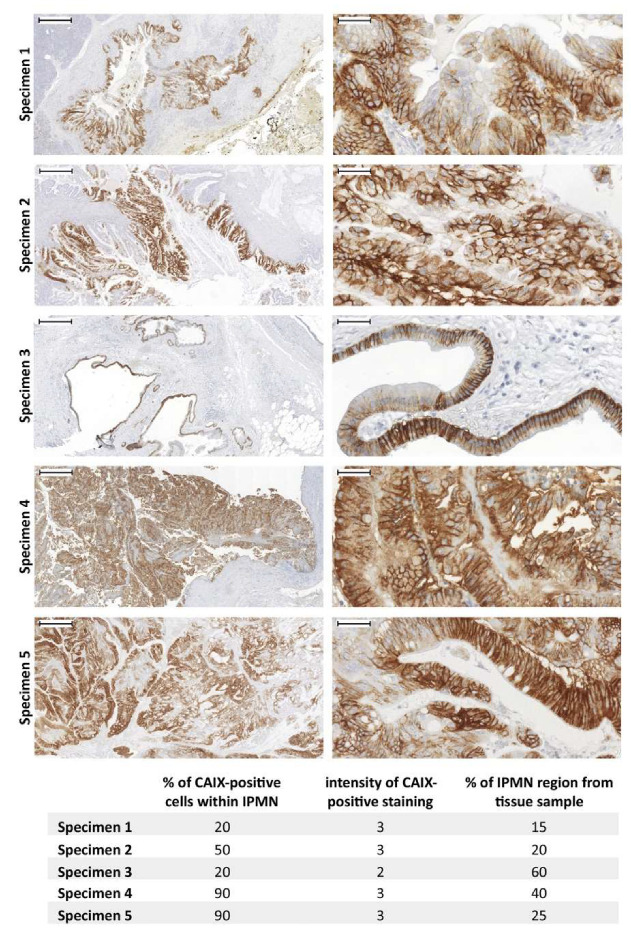
Immunohistochemical staining of CAIX in IPMN lesions within pancreatic ductal adenocarcinoma (PDAC) patient samples. The set of 5 IPMN tissue specimens were immunostained using the specific anti-CAIX monoclonal antibody M75 [143] as described previously [144] (Supplementary Material). Representative pictures taken from all 5 IPMN tissue specimens (left side) were complemented with detailed pictures (right side) describing CAIX-specific staining pattern (brown). All sections were counterstained with Mayer’s hematoxylin (blue nuclei). Scale bar 500 μm (left side) and 50 μm (right side).

However, the immunohistochemical staining of CAIX in IPMNs has never been outlined before. Thus, we were interested in the immunohistochemical evaluation of CAIX expression in IPMNs which were identified in 5 cases from the cohort of 55 PDAC patients (Supplementary Material). As shown in Figure 6, all 5 IPMN samples were strongly CAIX-positive (predominant value 3 from the scale 0-3) and the percentage of CAIX-positive cells within IPMN lesions ranged from 20% to 90% (see table within Figure 6). Additionally, the IPMN region proportion within each tissue sample was evaluated, with all data summarized in Figure 6. Based on multiple evidence (expanded upon in Section 2, which discusses transcriptional regulation of CA9 in pancreatic tumors), high CAIX-positivity within IPMN could be affected by the activity of several signaling pathways relevant to the microenvironment of precursor lesions.

Interestingly, expression of glycolysis-associated genes which reflect adaptation to hypoxia or Warburg effect phenotype of cancer cells, characteristic of a CAIX-related TME, are already overexpressed in PanIN and IPMN lesions. Immunostaining positivity of PKM2 progressively increases from PanIN to pancreatic cancer. LDHA is strongly upregulated even in pancreatitis and there are no significant differences between chronic pancreatitis, PanIN, and cancer samples [141]. Furthermore, a considerable increase in LDHA and MCT4 staining was also observed in patients with IPMN lesions, mainly in those diagnosed with high-grade IPMN who later (after surgical resection of IPMN lesion) progressed to adenocarcinoma or whose resected pancreas specimens already contained IPMN-associated adenocarcinoma, compared to patients with only benign IPMN [142].

**Figure 7:**
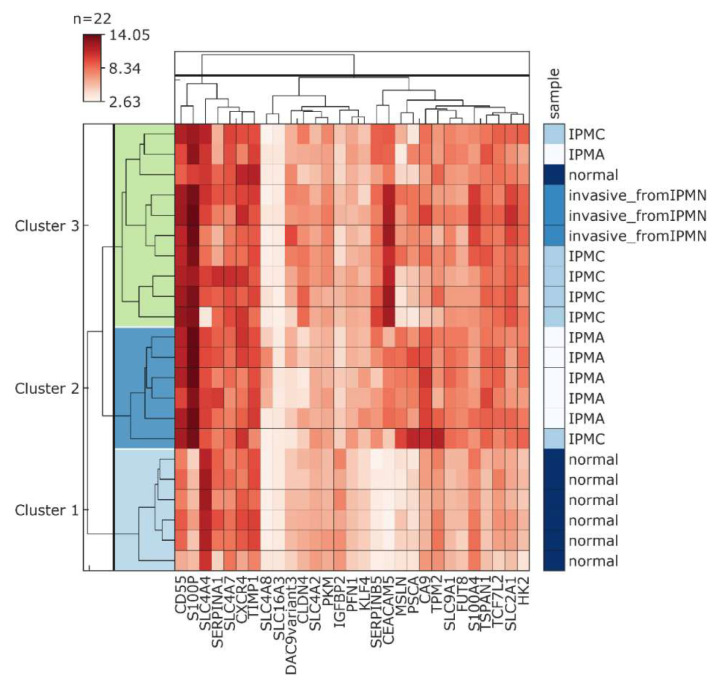
Hierarchical clustering of GSE19650 samples according to the panel of Genes Highly Expressed in IPMNs as Compared with Normal Pancreatic Ductal Epithelium and Genes Associated with Invasive Intraductal Papillary Mucinous Neoplasms of the Pancreas [139], selected pH regulators (SLC4A7, SLC4A4, SLC4A8, SLC4A2, SLC9A1), and glycolysis-related genes (HK2). Genes from the panel of Sato et al. (2004) as well as CA9 show a mutual correlation and a differential expression between normal healthy samples and IPMN samples. Hierarchical cluster analysis was able to correctly cluster the majority of normal healthy tissue samples into one cluster and all mucinous neoplasm samples were grouped into two clusters: cluster 2 contains mainly non-invasive adenoma samples, while cluster 3 includes the majority of adenocarcinoma with invasion and invasive carcinoma.

Using a large-scale profiling of gene expression among 12 intraductal papillary mucinous neoplasms with and without associated invasive carcinoma, Sato et al. identified a panel of genes significantly upregulated (fold change of 5 or more) in IPMN in comparison to healthy tissue samples and genes potentially linked to an invasive phenotype of IPMN [139]. Interestingly, multiple genes found among the most highly upregulated ones in IPMNs have been already reported as overexpressed in PDAC. Our in silico analysis of the GSE19650 dataset revealed that CA9 displays a high positive correlation with several of these genes (Figure 7), such as S100P (correlation coefficient 0.67), mesothelin MSLN (0.82), prostate stem cell antigen (PSCA) (0.82) and, to a lesser extent, with CD55 (0.46), TPM2 (0.35), and SERPINB5 (0.29). CA9 expression was also linked to glycolysis-related gene HK2 and genes coding proteins involved in pH regulation (anion exchanger 2 (AE2), sodium/hydrogen exchanger 1 (NHE1)).

**Figure 8:**
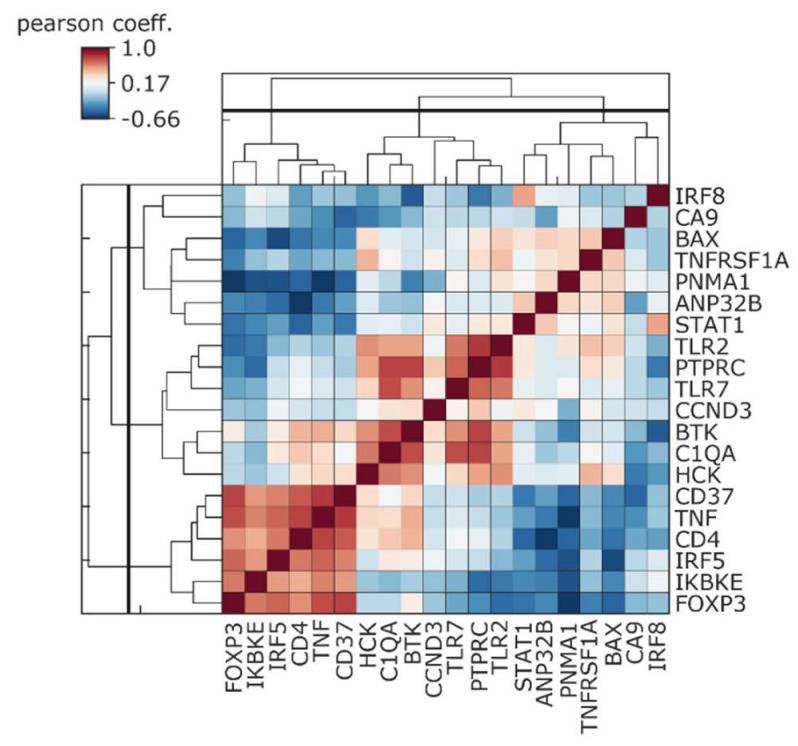
Negative correlation between CA9 and immune panel genes which are upregulated in samples with better prognosis. The analysis was performed with GSE19650 consisting of intraductal mucinous neoplasia and normal tissues. Immune gene panel indicates immune-related genes as predictors of outcome in pancreatic adenocarcinoma according to D’Angelo et al. [145].

We also evaluated a possible relationship between CA9 expression and expression of immunity-related genes which were significantly upregulated in pancreatic ductal adenocarcinoma patients with a good prognosis compared to patients with a worse prognosis [145]. Already in IPMN lesions, CA9 showed a varying degree of negative correlation, with the analyzed immune competent genes highly expressed in patients with a good outcome (Figure 8).

Notably, the TCGA analysis performed by McDonald et al. on 135 PDAC cases revealed that CAIX expression correlates with a set of genes associated with glycolysis including LDHA, monocarboxylate transporters (MCT4, MCT1), glucose transporters (GLUT1, GLUT4), and pyruvate dehydrogenase kinase 1 (PDK1) [75]. Patients with very low levels of CAIX and other selected hypoxia adaptation genes exhibited significantly longer survival compared to the group with high levels of expression. Based on tissue microarray staining, CAIX-positive tumors were identified in 66% of PDAC patients. Similarly, an association between CAIX and glycolysis was shown in breast cancer cells. Suppression of CAIX led to attenuation of glycolysis with reduction of PDK1 expression/function through LIN28/let-7 axis [146].

Interestingly, the pH-sensitive probe (pHLIP peptide) which was used to image PDAC xenografts was also able to detect PanIN lesions in genetically-engineered mouse models [147]. The pHLIPs are membrane peptides which insert themselves into the plasma membranes and form a transmembrane helix only within an acidic extracellular microenvironment. This is the first in vivo evidence of an acidic pancreatic cancer microenvironment even in pre-cancerous lesions. All these data suggest that CAIX could serve as a marker of pancreatic intraepithelial neoplasia. Importantly, another technique of imaging extracellular acidosis in the TME is acidoCEST-MRI (chemical exchange saturation transfer (CEST) magnetic resonance imaging) [148]. This non-invasive method, which uses a contrast agent and instrumentation routinely available in radiology clinics, can accurately measure tumor pHe with its spatial heterogeneity. AcidoCEST-MRI was successfully used to monitor changes in tumor acidosis of pancreatic cancer mice models. pHe significantly differed between the metformin-treated group and the control group due to the therapeutic efficiency of metformin, which counteracts glycolysis and thus pHe [148]. Furthermore, acidoCEST-MRI evaluating pHe in B-cell lymphoma xenografts showed strong correlation between extracellular acidity and CAIX expression [149]. Current progress in the in vivo measurement of microenvironmental acidosis of pancreatic adenocarcinomas is reviewed in [150].

Thus, whilst CAIX significantly contributes to extracellular acidosis of tumors, the imaging of tumor pHe in vivo using different pH-sensitive probes or by specific anti-CAIX antibodies could lead to early detection of pre-malignant lesions which are undetectable by commonly used imaging techniques. In fact, the chimeric anti-CAIX antibody cG250 was shown to excellently detect CAIX-positive primary renal ccRCC tumors and their metastatic lesions in the human body [151].

Importantly, recent data from mouse models of PDAC and human PanIN lesions indicate that hypoxic microenvironments emerge early during pancreatic carcinogenesis. HIF-1α and hypoxyprobe positivity was observed even in sporadic PanIN lesions from 2-month old K-rasG12D mice (p48-Cre; LSL-K-rasG12D autochthonous PDAC mouse model) without developed adenocarcinoma [152]. Nuclear HIF-1α staining was also confirmed in PanINI to PanINIII lesions from human samples. These data suggest that CAIX expression, along with hypoxia response and acidosis, play a role in the early phase of pancreatic carcinogenesis.

## 6. Influence of CAIX Expression and CAIX-Associated Tumor Microenvironment on Therapy Resistance Explored in Preclinical Models of Pancreatic Cancer

Poor response to anti-cancer therapies remains one of the main problems of pancreatic cancer. Hypoxia—in adverse feature of pancreatic cancer—mediates resistance to chemotherapy, radiotherapy, and immunotherapy, and creates the niche supporting the stemness of tumor-initiating cells (TICs).

Cooperation between hypoxia stimuli and paracrine signaling from an inflammatory environment, an inherent feature of pancreatic tumors, induces EMT, stemness, and promotes the formation of circulating tumor cells [126]. High CAIX expression was detected in tumor-initiating cells (TICs) positive for pancreatic cancer stem cell markers EpCAM^+^/CD44^+^/CD24^+^, isolated from PDAC patient samples [29]. Silencing of CA9 in the EpCAM^+^/CAIX^high^ TICs population resulted in failed tumor initiating activity in mice xenografts. These data indicate that inhibition of CAIX with specific antibodies or inhibitors may impair tumor growth and metastasis.

Shukla et al. showed that HIF-1α-dependent high glycolytic flux is responsible for gemcitabine resistance in pancreatic cancer cells [153]. The mechanism behind this phenomenon is molecular competition between an increased cytidine pool and gemcitabine, which cannot be efficiently incorporated into the replicating DNA. HIF-1α target genes, TKT and CTPS1, regulating pyrimidine synthesis are associated with primary and metastatic pancreatic cancer tissues. Importantly, inhibition of HIF-1α—pharmacologically or genetically—improves gemcitabine sensitivity even in patient-derived xenografts (PDXs) in nude mice. TKT and CTPS1 co-localized with CAIX in PDAC tissue samples and in orthotopic pancreatic tumors. The authors hypothesize that hypoxia plays an important role in gemcitabine resistance in vivo through the modulation of TKT, CTPS1, and other glycolytic enzyme expression, whilst CAIX is the component of this microenvironment. Co-expression of CAIX with HK2, a surrogate marker of HIF-1α activation, was detected in orthotopic tumors derived from isolated mouse PDAC cell lines, which histologically resembled human hypoxic/necrotic PDAC phenotype including hypovascularization.

Similarly to HIF-1α, oncogenic K-ras plays a key role in the upregulation of glycolysis in PDACs through the transcriptional regulation of glucose transporters, monocarboxylate transporters, and glycolytic enzymes. Many glycolysis-related genes are overexpressed and are associated with poor prognosis of PDAC (e.g., LDHA, MCT4, GLUT1, HK2). An in silico analysis by McDonald and colleagues revealed the association of high CAIX expression with glycolytic phenotype (GLUT1, LDHA, MCT4, MCT1, PDK1) and poor prognosis of PDAC patients [75]. They also showed that CAIX is highly expressed in K-ras-driven KPCY (K-rasG12D/Pdx1-Cre/p53/RosaYFP) pancreatic tumors in genetically-engineered mouse models (GEMM). Silencing of CAIX in cell lines derived from KPCY GEMM tumors, implanted orthotopically, reduces tumor burden, and metastatic dissemination. Importantly, the administration of CAIX-specific inhibitor SLC-0111 to mice bearing PDAC xenografts reduced tumor growth compared with gemcitabine alone, while increasing their sensitivity to gemcitabine and significantly prolonging survival. Combined therapy also dramatically improved survival in orthotopic patient-derived xenograft (PDX) models where, after 3 months, 100% of cases were alive compared to around 70% in the gemcitabine-alone group, and none in the vehicle group. Furthermore, recent evidence indicates that CAIX enzymatic activity and associated extracellular acidosis play a key role not only in survival of cancer cells in hypoxia and their migration/invasion properties, but also in immunosurveillance. Antitumor immunity is attenuated by highly-acidic TME via several mechanisms [154,155,156]. For example, low pH inhibits infiltration and cytolytic activity of CD8^+^ T lymphocytes, but does not affect Treg cells which are immunosuppressive. Other mechanisms include inhibition of cytokine production, macrophage switch from M1 immune competent to M2 immunosuppressive phenotype, or resistance to immune check point immunotherapy [157].

**Figure 9:**
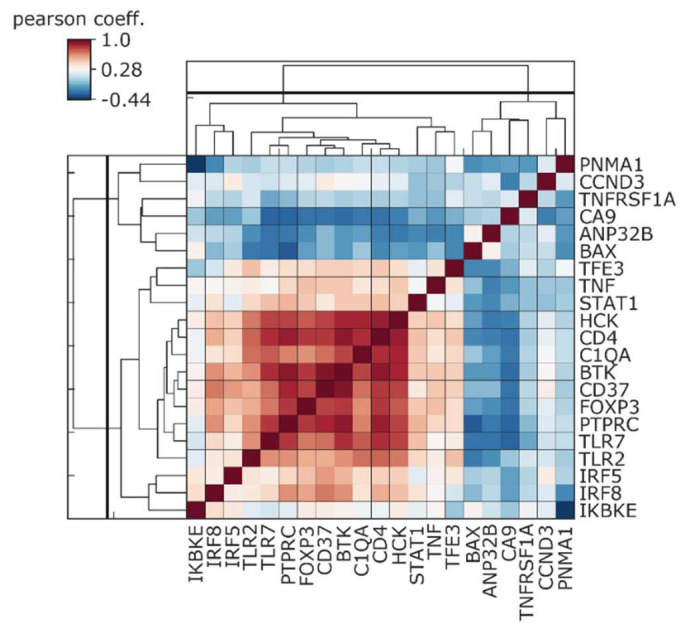
Negative correlation between CA9 and immune panel genes which are upregulated in samples with better prognosis. The analysis was performed with a cohort of 183 PDAC samples from TCGA (Xena).

Neutralization of tumor acidity, which is known to inhibit tumor growth or metastasis of some cancer types [158,159], is also associated with increased CD8^+^ T-cell infiltration in immunocompetent mice [157]. A combination of neutralization of microenvironmental acidosis with anti-CTLA4 or anti-PD1 immune check point antibody greatly improved the anti-tumor response of these therapies. Pharmacological inhibition of tumor acidosis represents a promising tool to treat different cancers and overcome their metastatic spread. Orthotopic models of Panc02 human pancreatic cancer cells were insensitive to anti-PD-1 antibody as a monotherapy, but in combination with a specific CAIX inhibitor (DH348) or LDHA inhibitor (FX-11), were efficient in overcoming resistance to PD-1 blockade, while constraining metastasis [158]. The combination of SLC-0111 with anti-PD1 and anti-CTLA4 in mice bearing orthotopic breast tumors did not lead to visible tumor growth delay. However, histological evaluation showed increased necrosis, the extent of which was almost doubled in tumors treated with triple combination compared to tumors treated with SLC-0111 alone or an anti-PD1/anti-CTLA4 combination, respectively [160]. Moreover, triple combination treatment or SLC-0111, combined with either ICP antibody separately, achieved the most effective reduction of lung metastatic burden. Immune profiling of collected tumors revealed depletion of immune suppressive Tregs and Th17 cells, and a rise in cytotoxic CD8^+^ T-cells and Th1 cells, within the tumors treated with all three agents [160]. Thus, TME with decreased acidosis through CAIX inhibition creates a supportive environment for the effective activation of anti-tumor immunity, including efficiency of ICB immunotherapy. We assessed CA9 expression and the immune gene panel identified by D’Angelo et al. [145], differentiating patients with good and worse prognosis in a cohort of 183 PDAC samples from the TCGA database. In silico analysis revealed a negative correlation between CA9 and genes which are highly expressed in patients with a good outcome (Figure 9). Similarly, inverse correlation of CA9 with an immune activity signature was described for melanoma and basal-like breast cancer patients [160].

Alternatively, inhibition of metastasizing through the targeting of acidosis using DH348, acetazolamide, FX-11, and bicarbonate was also able to effectively reduce metastasis arising after tail vein injection in mice [158]. Additionally, specific anti-CAIX antibodies prevented lung metastasis formation of intravenously injected HT1080 cells in mice. Moreover, these antibodies also abrogated the capability of cancer cells to invade through the chorioallantoic membranes of quail embryos [16].

## 7. Clinical Trials Targeting CAIX-Related Molecular Pathways

The prevention, diagnosis, and treatment of pancreatic cancer are extremely difficult as symptoms are non-specific or even lacking, and effective screening tools for detection are not in place. Once diagnosed, the disease has usually advanced, making fewer than 20% of patients fit candidates for surgical resection [161]. Therapeutic options are limited, as most pancreatic cancers—owing to their complex genetic and metabolic nature and the ongoing crosstalk between the cells of the TME—have a poor response to chemotherapy. This naturally translates to an extremely dismal mortality–incidence ratio of 94% [32]. With 2671 currently registered clinical trials at https://clinicaltrials.gov, the devastating fatality of pancreatic cancer undoubtedly drives further development of effective therapeutic protocols. Here, we summarize completed, ongoing, and recruiting clinical trials in pancreatic cancer targeting CAIX-related molecular pathways, while still relying on treatment “backbones” most frequently consisting of gemcitabine, nab-paclitaxel, or the combination chemotherapy regimen FOLFIRINOX (oxaliplatin, irinotecan, fluorouracil, and leucovorin) now considered the standard first-line treatment for patients diagnosed with advanced stage pancreatic cancer, as it provides a 4.3 month increase in OS when compared to gemcitabine alone [162].

### 7.1. CAIX

SLC-0111-mediated CAIX inhibition has proven to be effective in patient-derived 2D and 3D PDAC cell cultures [64,65], followed by an SLC-0111 safety study (NCT02215850) in advanced solid tumors. Currently, a Phase 1/2 clinical trial (NCT03450018) uses the selective, small molecule inhibitor SLC-0111 in CAIX-positive patients diagnosed with metastatic PDAC. The study completion is estimated in 2022. CAIX is actively involved in the metastatic cascade and migration [12], localizes at focal adhesion sites [23], and within invadopodia [16]. A currently-recruiting Phase 1 clinical trial (NCT03199586) aims to target the migratory pathway via NP-G2-044. This drug targets and binds fascin and, hence, obstructs the fascin/actin interaction. By preventing filopodia formation and cytoskeletal reorganization, NP-G2-044 impairs cancer cell migration.

### 7.2. Angiogenesis

Angiogenesis in solid tumors is regulated by hypoxia and acidic pH. VEGF expression is under the regulation of HIF [163], while CAIX expression supports angiogenesis by contributing to extracellular acidosis [21]. In line with this, the inhibition of CAIX was shown to significantly enhance anti-VEGF therapy in a preclinical setting [164]. Patients with increased tissue and plasma CAIX values are unlikely to profit from sunitinib-based anti-angiogenic therapy [165]. Combination therapy of the recombinant humanized anti-VEGF monoclonal antibody bevacizumab and gemcitabine in a Phase 2 clinical trial yielded a partial response for 21% of patients and disease stabilization in 46% of patients diagnosed with advanced pancreatic cancer [166], with a significantly decreased reciprocal of doubling time [167] reported in another study (NCT00460174). The combination of bevacizumab, capecitabine, and gemcitabine (NCT00100815) results in a mean progression free survival (PFS) of 5.7 months and a mean OS of 9.8 months, but a regimen consisting of bevacizumab, capecitabine, and radiation therapy still seems to be more efficient with a reported median PFS of 8.6 months and a median survival rate of 11.9 months (NCT00114179; [168]).

While targeting VEGF in pancreatic cancer might not result in the anticipated outcome, preventing neo-vascularization by anti-angiogenic therapy still remains a promising way of preventing tumor growth. A new antiangiogenic drug combination TL-118 was reported to be extremely effective in a case report study [169]. When re-administered, the drug efficiently decreased elevated tumor marker levels observed after TL-118 treatment termination. The studied pancreatic cancer patient, treated with standard chemotherapy and TL-118, was still considered progression-free 16 months post-diagnosis. Two Phase 2 clinical trials (NCT01509911, NCT01659502) studying TL-118 in pancreatic cancer followed.

Clinical studies have also examined the effect of multi-targeted tyrosine kinase inhibitors, e.g., sunitinib malate (NCT00397787, NCT00462553, NCT00967603), axitinib (NCT00471146), masitinib (NCT00789633), and vatalanib (NCT00185588). Sunitinib [170] and axitinib [171] seem to have minimal activity in advanced pancreatic adenocarcinoma. A clinical trial studying the effects of masitinib in combination with gemcitabine divided patients into two sub-groups based on acyl–CoA oxidase-1 (ACOX1) overexpression or baseline pain intensity threshold evaluation. While patients in the latter study group achieved a mean OS of 8 months, masitinib treatment resulted in significantly increased OS in the ACOX1 group (median 11.7 months; [172]). The combined Phase 1/2 clinical trial (NCT00185588) examines the drug combination of vatalanib and gemcitabine for patients with unresectable pancreatic cancer. After successful optimal safe and tolerable dose determination, the antitumor efficacy of this regimen is to be established.

### 7.3. Epidermal Growth Factor Receptor (EGFR) Inhibition

EGFR activates the MAPK/ERK and PI3K/AKT pathways, both involved in the transcriptional activation of CA9 expression [61,62]. Two Phase 1 clinical trials (NCT04045496) are yet to investigate the MAPK inhibitor JAB-3312 in advanced solid tumors, including pancreatic cancer. The MEK kinase inhibitor AZD6244 was evaluated in comparison to capecitabine in an already-completed Phase 2 clinical trial (NCT00372944) designed for patients suffering from pancreatic cancer for whom gemcitabine therapy has failed. The anti-EGFR monoclonal antibody cetuximab was investigated in numerous clinical trials, including a Phase 2 clinical trial (NCT00225784) where the drug was administered in combination with gemcitabine and radiotherapy. Out of 33 participants, 10 partial responses were achieved, the disease was stabilized in 20 patients and, in 3 cases, the disease progressed. However, cetuximab-mediated EGFR inhibition does not seem to be beneficial when compared to cetuximab-free study arms (NCT00042939, NCT00075686, NCT00536614). The consensus among clinical studies and literature is that cetuximab does not increase response nor survival in EGFR-positive patients [173] and does not yield an improved outcome compared to gemcitabine [174]. The EGFR inhibitor nimotuzumab was investigated in a Phase 2/3 clinical trial (NCT00561990), where the combination of gemcitabine and nimotuzumab was shown to be well-tolerated and significantly improved the 1-year OS, with a significantly better OS (11.6 versus 5.6 months, *p* = 0.03) achieved in patients harboring the K-ras mutation [175]. The drug erlotinib, responsible for inhibiting EGFR phosphorylation and subsequent signalization, was investigated in a Phase 2 clinical study (NCT00810719) in combination with gemcitabine for treating patients with metastatic/recurrent pancreatic cancer, but showed no signs of efficacy. Another clinical trial (NCT01608841) analyzing the same drug combination showed gemcitabine + erlotinib to have a superior efficacy compared to gemcitabine alone. Disease control (including complete response, partial response, and stable disease) in metastatic pancreatic cancer patients was significantly better (85% vs. 33%; *p* = 0.001) in patients harboring the EGFR mutation [176]. A Phase 3 clinical trial (NCT00040183) with 569 patients enrolled also reported results in favor of the erlotinib/gemcitabine treatment, indicating a longer 1-year survival when erlotinib was administered (23% v 17%; *p* = 0.023). The achieved OS was also statistically significant but still extremely modest (6.24 months vs. 5.91 months), considering that patients in the erlotinib arm suffered from more grade 1 and 2 adverse events [177].

The combination of bevacizumab and erlotinib was shown to be inefficient for patients resistant to gemcitabine (NCT00365144, NCT00366457, NCT00925769), as the overall 6-month survival rate was only 22% [178]. A clinical trial (NCT00091026) also compared the effects of bevacizumab and gemcitabine with either cetuximab or erlotinib, but no difference between mean PFS and OS was observed between study arms. Similarly, the difference in 2-year OS and disease-free survival between cetuximab and bevacizumab administered as a complement to gemcitabine, capecitabine, and radiation is statistically insignificant among study arms (NCT00305877).

### 7.4. AKT

Inhibition of the AKT signaling pathway can clinically be achieved by MK-2206, which inhibits the kinase in an ATP-noncompetitive manner, and RX-0201, an Akt1 antisense oligonucleotide. A preclinical study evaluating the combination of the CDK inhibitor dinaciclib and MK-2206 showed great promise in pancreatic cancer therapy, as treatment achieved some complete responses and blocked tumor growth in all of the patient-derived xenograft models [179]. Based on these results, a clinical trial (NCT01783171) was initiated. However, MK-2206 administered in combination with selumetinib showed no advantage for patients for whom gemcitabine-based therapy had failed (NCT01658943). A Phase 2 clinical trial (NCT01028495) using RX-0201 + gemcitabine for patients with metastatic disease was initiated based on a preclinical study on pancreatic cancer cell lines and xenograft mouse models, which showed the therapeutic potential of the novel Akt1 antisense oligonucleotide [180].

### 7.5. Hypoxia-Activated Prodrugs

Hypoxic regions of solid tumors frequently account for treatment failure and, hence, are an attractive target for novel drug design and therapy. TH-302 (evofosfamide), an investigational hypoxia-activated prodrug, is reduced by one-electron reductive enzymes (e.g., NAPDH cytochrome P450) to form a radical anion. Under hypoxic conditions, it is further fragmented, generating bromo-isophosphoramide mustard (Br-IPM). Hypoxia-dependent TH-302 activation results in a cytotoxic agent mediating DNA crosslinking and cell cycle arrest [181]. Several clinical trials have evaluated the effects of TH-302 in combination with gemcitabine and/or nab-paclitaxel. In a preclinical study [182], the combination of gemcitabine + nab-paclitaxel + TH-302 has shown superior efficacy in human tumor xenograft models of pancreatic cancer. These promising results translated into a Phase I clinical trial (NCT02047500), which has unfortunately been terminated following the company’s decision to discontinue the clinical development of evofosfamide. In a conducted Phase 2 study (NCT01144455), gemcitabine + TH-302 yielded a significantly longer PFS and an improved tumor response [183] compared to gemcitabine alone. The drug combination was further investigated in a Phase 3 clinical trial (NCT01746979).

### 7.6. Hedgehog Signaling

The hypoxia-triggered SHH pathway [85] is aberrantly activated in a malignant pancreas [184]. Since HH signaling inhibition enhanced gemcitabine delivery in mice [185], targeting the hedgehog pathway in pancreatic cancer became very promising. Vismodegib (GDC-0449), a selective HH pathway inhibitor which binds to the SMO receptor, repeatedly did not show improved PFS and OS in Phase 1/2 clinical trials (NCT01064622, NCT01088815, NCT01195415) and was not shown to be superior to gemcitabine alone in the treatment of metastatic pancreatic cancer. LDE-225, an SMO antagonist, was also shown to inhibit EMT [186] and, combined with gemcitabine and nab-paclitaxel, was proven to be well tolerated (NCT02358161) with promising efficacy [187]. An additional SMO inhibitor compound, IPI-926, has also been tested in several Phase 1/2 clinical trials combined with gemcitabine (NCT01130142; [188]).

### 7.7. Mammalian Target of Rapamycin (mTOR)

The mammalian target of rapamycin (mTOR) is an upstream regulator of HIF-1α [189], as well as of a number of other downstream target genes involved in angiogenesis, cell growth and proliferation, bioenergetics, and survival [190]. As such, it is another rational target in the treatment of pancreatic cancer [191]. CAIX knockdown was shown to improve the therapeutic efficacy of rapamycin preclinically [192], justifying clinical evaluation of such dual targeting. Drugs targeting the mTOR pathway that are currently under clinical investigation for the treatment of pancreatic cancer include sirolimus (rapamycin) and its analogue everolimus. A Phase 1/2 clinical study (NCT03662412) aims to investigate the effect of sirolimus monotherapy and is currently recruiting participants, with an estimated study completion in 2023. A Phase 3 clinical trial (NCT00510068) evaluated the efficacy of everolimus in pNET patients, with an encouraging median PFS of 11.04 months for patients treated with everolimus compared to a median PFS of 4.60 for patients treated with a placebo. Despite being statistically insignificant, the OS in this study was also promising—everolimus treatment yielded a median OS of 44.02 months (max. 51.75 months), while placebo treatment resulted in an OS median of 37.68 months. The safe and well-tolerated anti-diabetic drug metformin was shown to efficiently suppress mTOR activation [193] in pancreatic cancer [194], as well as other cancers [195]. In a clinical setting, the combination therapy consisting of gemcitabine, erlotinib, and metformin had no effect on the outcome of patients suffering from advanced pancreatic cancer when compared to the placebo (NCT01210911; [196]). In patients diagnosed with diabetes mellitus prior to pancreatic cancer diagnosis, metformin use translated into an improved outcome in patients suffering from non-metastatic disease, suggesting an improved chemotherapy response [197].

### 7.8. Immunotherapy

Aiming to boost the function of the immune system and elicit an anti-tumor response via T-cell activation, a number of agents targeting immune checkpoint inhibitors of the PD-1/PDL-1 pathway are already approved by food and drug administration (FDA) for different cancer types [198,199]. Immune activity is hindered by low pH [157,200] and lactate accumulation [201], providing a rationale for combining CAIX inhibition with immune checkpoint blockade-based therapy in a clinical setting. In support of this, Chafe et al. showed that by reducing pHe acidification, SLC-0111 enhances T-cell killing and aids anti-PD1 therapy in vitro [160]. Most clinical trials at various stages targeting PD-1 in pancreatic cancer are currently recruiting or ongoing (Table 1), including a Phase 1 clinical trial (NCT04181645) with an estimated termination date in 2022 evaluating the safety and efficacy of the combination treatment of SHR-1210 (a humanized anti-PD1 monoclonal antibody), nab-paclitaxel, and gemcitabine in a cohort of metastatic PDAC patients. A Phase 3 clinical trial of 830 participants (NCT03983057) is currently investigating antibody-mediated PD1 inhibition together with chemotherapy in relation to response rate, OS, and PFS.

## 8. Conclusions and Future Directions

Currently, the most common diagnostic tools to detect PDAC are imaging techniques including computed tomography (CT), magnetic resonance imaging (MRI), positron emission tomography (PET), and endoscopic ultrasound (EUS), correlated with blood levels of CA19-9 and amylase. Unfortunately, they are unable to detect early lesions and are mainly used for disease staging.

Features of the pancreatic cancer TME such as desmoplasia, hypovascularization, hypoxia, and acidosis represent a physical-ecological barrier implicated in resistance to chemotherapy, radiotherapy, and immunotherapy. As we summarize in this review, even the early stages of pancreatic carcinogenesis are associated with hypoxia response, acidosis, and CAIX expression, which are exploitable diagnostic targets. Extensive data indicate that CAIX is a valuable diagnostic marker and therapy target. Detection of acidic TME through pH-sensitive probes or specific anti-CAIX antibodies, conjugated with agents detectable by routine radiomic imaging used in pancreatic cancer diagnostics, could represent a promising tool for identification of early pre-cancerous lesions. Indeed, an ^131^I-cG250 conjugated antibody-recognizing CAIX protein was successfully used in clinics to detect CAIX-positive primary tumor and disseminated metastasis [151]. Animal models show that CAIX can be a useful target for molecular imaging based on monoclonal antibodies or inhibitors tagged with various radionuclides [215,216,217].

There is an ongoing effort to identify parameters and novel predictive markers which would help to tailor therapy for pancreatic cancer patients as well as to predict treatment outcome. Recent studies investigated the possibility of predicting the response to neoadjuvant radiochemotherapy according to microenvironmental factors (hypoxia, tumor vascularization, stromal activation) and evaluated hypoxia as a relevant tool for patient stratification. Studies have used pre-operative pimonidazole administration correlated with post-operative CAIX staining as a means to determine hypoxia extent (NCT01989000, NCT03718650, NCT01248637). One of the aims of these studies is to assess if features of non-invasive radiomic imaging prior to surgery correspond with intratumor hypoxia determined in the specimen after surgery by immunohistochemical staining of CAIX, GLUT1, or HIF-1α.

Since hypoxia and acidosis strongly influence anti-tumor treatment efficacy, targeting hypoxic/acidic TME through CAIX protein can enhance the efficiency of conventional therapies and improve the infiltration of immunocompetent cells or overcome immunotherapy resistance. Increased efficiency of combinatorial treatment consisting of CAIX inhibition and clinically-used therapies or those under clinical trials in PDAC patients was shown in different preclinical pancreatic or other models. This includes the knockdown/inhibition of CAIX combined with the chemotherapy drugs gemcitabine or paclitaxel, mTOR inhibitors (rapamycin), inhibitor of HIF-1α-targeting APE1/Ref-1 (APX3330), inhibition of angiogenesis (bevacizumab), or in combination with immune check point immunotherapy [65,75,158,160,164,192,218]. Furthermore, data from a Phase 3 clinical trial (ARISER) using a monoclonal antibody against CAIX, cG250, where the mechanism of action is based on immune response (antibody-dependent cellular cytotoxicity), revealed a prolonged disease-free survival up to 22 months in a patient cohort with high CAIX [219].

In conclusion, based on vast evidence from literature and clinical trials, we believe that therapies combining clinically-administered therapeutic regimens with CAIX targeting could improve the efficacy of anti-tumor response in patients with pancreatic ductal adenocarcinoma.

## Figures and Tables

**Table 1:** Clinical trials for pancreatic cancer targeting molecular pathways with relationship to CAIX.

	Identifier	Target	Agent	Treatment “Backbone”	Phase	Status	Ref.
**CA IX**	NCT03450018	CA IX	SLC-0111	Gemcitabine	1/2	Recruiting	
**Migration**	NCT03199586	Fascin	NP-G2-044		1	Recruiting	
**Angiogenesis**		VEGF	Bevacizumab	Gemcitabine	2	Completed	[166]
	NCT00460174	VEGF	Bevacizumab	Gemcitabine and radiation therapy	2	Completed	[167]
	NCT00126633	VEGF	Bevacizumab	Gemcitabine and cisplatin	2	Completed	[202]
	NCT00100815	VEGF	Bevacizumab	Gemcitabine and capecitabine	2	Completed	
	NCT00114179	VEGF	Bevacizumab	Gemcitabine	2	Completed	[168]
	NCT00088894	VEGF	Bevacizumab	Gemcitabine	3	Completed	
	NCT00091026	VEGF, EGFR	Bevacizumab + Cetuximab/Erlotinib	Gemcitabine	2	Completed	
	NCT00365144	VEGF, EGFR	Bevacizumab + Erlotinib		2	Completed	[178]
	NCT00305877	VEGF, EGFR	Bevacizumab/Cetuximab	Gemcitabine, capecitabine, and radiation	2	Completed	
	NCT01509911	-	TL-118	Gemcitabine	2	Unknown	
	NCT01659502	-	TL-118		2	Unknown	
	NCT00397787	multi TKI	Sunitinib		2	Completed	[170]
	NCT00462553	multi TKI	Sunitinib	Gemcitabine	1	Completed	
	NCT00967603	multi TKI	Sunitinib		2	Completed	
	NCT00789633	multi TKI	Masitinib	Gemcitabine	3	Completed	[172]
	NCT00471146	multi TKI	Axitinib	Gemcitabine	3	Completed	[171]
	NCT00185588	multi TKI	Vatalanib	Gemcitabine	1|2	Completed	
**EGFR**	NCT00536614	EGFR	Cetuximab	Gemcitabine and cisplatin	2	Completed	[173]
	NCT00042939	EGFR	Cetuximab	Irinotecan and docetaxel	2	Completed	[203]
	NCT00225784	EGFR	Cetuximab	Gemcitabine and radiation therapy	2	Completed	
	NCT00075686	EGFR	Cetuximab	Gemcitabine	3	Completed	[174]
	NCT00561990	EGFR	Nimotuzumab	Gemcitabine	2|3	Completed	[175]
	NCT00810719	EGFR	Erlotinib	Gemcitabine	2	Completed	[204]
	NCT01608841	EGFR	Erlotinib	Gemcitabine	2	Unknown	[176]
	NCT00040183	EGFR	Erlotinib	Gemcitabine	3	Completed	[177,205]
	NCT01214720	VEGF, EGFR	Erlotnib ± Bevacizumab	Gemcitabine	3	Completed	
**MAPK/ERK**	NCT04045496	MAPK	JAB-3312		1	Recruiting	
	NCT00372944	MEK	Selumetinib	vs. Capecitabine	2	Completed	
**AKT**	NCT01783171	AKT, CDK	MK-2206 + Dinaciclib		1	Completed	[179]
	NCT01658943	AKT	MK-2206 + Selumetinib	vs. mFOLFOX	2	Completed	[206]
	NCT01028495	AKT	RX-0201	Gemcitabine	2	Completed	[180]
**Hypoxia**	NCT02047500	Hypoxia	Evofosfamide	Gemcitabine and nabpaclitaxel	1	Terminated	[182]
	NCT01144455	Hypoxia	Evofosfamide	Gemcitabine	1|2	Completed	[183]
	NCT01746979	Hypoxia	Evofosfamide	Gemcitabine	3	Completed	
**Hedgehog**	NCT01088815	Hedgehog	Vismodegib	Gemcitabine and nabpaclitaxel	2	Completed	[207]
	NCT01195415	Hedgehog	Vismodegib	Gemcitabine	2	Completed	[208]
	NCT01130142	Hedgehog	IPI-926	Gemcitabine	1|2	Completed	
	NCT02358161	Hedgehog	LDE-225	Gemcitabine and nabpaclitaxel			
	NCT01064622	Hedgehog	Vismodegib	Gemcitabine	1|2	Completed	[209]
	NCT01485744	Hedgehog	LDE-225	FOLFIRINOX	1	Active, not recruiting	
		Hedgehog	IPI-926	Gemcitabine	1		[210]
		Hedgehog	IPI-926	Gemcitabine	1|2		[188]
		Hedgehog	Vismodegib	Gemcitabine	1		[208]
**mTOR**	NCT01077986	mTOR, EGFR	Everolimus + Cetuximab	Capecitabine	1|2	Completed	
	NCT03662412	mTOR	Sirolimus		1|2	Recruiting	
	NCT01210911	mTOR, EGFR	Metformin + Erlotinib	Gemcitabine	2	Completed	[196]
	NCT03065062	mTOR, CDK4/6	Gedatolisib + Palbociclib		1	Recruiting	
	NCT02048384	mTOR	Metformin ± Rapamycin		1	Completed	
	NCT02978547	mTOR	Metformin		2	Not yet recruiting	
	NCT00075647	mTOR	Temsirolimus		2	Completed	
	NCT00510068	mTOR	Everolimus		3	Completed	[211,212,213]
**Immunotherapy**	NCT04377048	PD1	Nivolumab	Gemcitabine	2	Not yet recruiting	
	NCT02331251	PD1	Pembrolizumab	Gemcitabine and nabpaclitaxel	1|2	Terminated	[214]
	NCT02546531	PD1, FAK	Pembrolizumab + Defactinib	Gemcitabine	1	Active, not recruiting	
	NCT03983057	PD1	anti-PD1 Ab		3	Recruiting	
	NCT04212026	PD1	Nivolumab	IRE	2	Recruiting	
	NCT03716596	PD1	anti-PD1 Ab	SBRT	1	Recruiting	
	NCT03977272	PD1	anti-PD1 Ab	mFOLFIRINOX	3	Recruiting	
	NCT04181645	PD1	SHR-1210	Gemcitabine and nabpaclitaxel	1	Recruiting	
	NCT03374293	PD1	anti-PD1 Ab	Radiation	2	Recruiting	

## Supplementary Materials

The following are available online at https://www.mdpi.com/2072-6694/12/8/2005/s1. The detailed description of methods used in in silico analysis and immunohistochemistry is provided in Supplementary data file which is available online.

## Abbreviations

CEST	Chemical exchange saturation transfer
ACOX1	Acyl–coA oxidase-1
AP	Activating protein
APE1/Ref-1	AP (apurinic/apyrimidinic) endonuclease1/redox effector factor 1
ARG2	Arginase II
ATDC	Ataxia-telangiectasia group D complementing gene
CA	Carbonic anhydrase
CAF	Cancer associated fibroblast
CD	Cluster of differentiation
COX-2	Cyclooxygenase-2
CT	Computed tomography
CTLA4	Cytotoxic T-lymphocyte antigen 4
CTPS1	Cytidine 5’-triphosphate synthetase
DAMP	Damage-associated molecular pattern
DFS	Disease free survival
DNMT3a	DNA methyltransferase 3a
DVL-2	Dishevelled 2
EGFR	Epidermal growth factor Receptor
EMT	Epithelial–mesenchymal transition
EpCAM	Epithelial cell adhesion molecule
ERK	Extracellular signal-regulated kinase
FZD1	Frizzled-1
GEMM	Genetically engineered mouse model
GLI1	Glioma-associated oncogene homolog 1 (zinc finger protein)
GLUT	Glucose transporter
GTEx	Genotype-tissue expression
Her-2	Human epidermal growth factor receptor 2
HH	Hedgehog
HIF	Hypoxia-inducible factor
HK2	Hexokinase 2
HRE	Hypoxia-responsive element
IGF2BP3	Insulin-like growth factor 2 mRNA-binding protein 3
IL	Interleukin
IPMN	Intraductal papillary mucinous neoplasm
IQGAP1	IQ motif containing GTPase activating protein 1
K-ras	Kirsten rat sarcoma viral oncogene homolog
KIF14	Kinesin family member
LDHA	Lactate dehydrogenase A
MAPK	Mitogen-activated protein kinase
MCN	Mucinous cystic neoplasm
MCT	Monocarboxylate transporters
MEK	Mitogen-activated protein kinase
MRI	Magnetic resonance imaging
mTOR	Mammalian target of rapamycin
MUC	Mucin
MVD	Microvessel density
MVI	Microvessel integrity
NBCe1	Electrogenic sodium/bicarbonate cotransporter 1
NBCn1	Electroneutral sodium/bicarbonate cotransporter 1
NF-κB	Nuclear factor κappa-light-chain-enhancer of activated B cells
NHE1	Sodium/hydrogen exchanger 1
OS	Overall survival
PanIN	Pancreatic intraepithelial neoplasia
PD1	Programmed cell death protein 1
PDAC	Pancreatic ductal adenocarcinoma
PDK1	Pyruvate dehydrogenase kinase 1
PDL-1	Programmed death-ligand 1
PDX	Patient-derived xenograft
PET	Positron emission tomography
PFS	Progression-free survival
PG	Proteoglycan-like domain
PGE2	Prostaglandin E2
pHLIP peptide	pH (low) insertion peptide
PI3K	Phosphatidyl inositol 3-kinase
PKM2	Pyruvate kinase muscle isozyme
PPBP	Pro-platelet basic protein
PSCA	Prostate stem cell antigen
qRT-PCR	Real-time quantitative reverse transcription PCR
RTK	Receptor tyrosine kinase
SERPINB5	Serpin family B member 5
SHH	Sonic Hedgehog
SLC	Solute carrier family membrane transport protein
SMO	Smoothened
SP1	Specificity protein 1
TCGA	The cancer genome atlas
TET	Ten-eleven translocation enzymes
TF	Transcription factors
TGF-β	Transforming growth factor β
Th cells	T-helper cells
TIC	Tumor initiating cell
TKT	Transketolase
TLR	Toll-like receptor
TME	Tumor microenvironment
TMPRSS3	Transmembrane serine protease 3
TNF-α	Tumor necrosis factor α
TPM2	β-tropomyosin
Tregs	T regulatory cells
TSS	Transcription start site
uPAR	Urokinase-type plasminogen activator receptor
VEGF	Vascular endothelial growth factor
α-SMA	α-smooth muscle actin
