# Supplementary material for: Clinical and Pre-Clinical Evidence of Carbonic Anhydrase IX in Pancreatic Cancer and Its High Expression in Pre-Cancerous Lesions
Source: Cancers (Basel). 2020 Jul 22;12(8):2005. doi: 10.3390/cancers12082005 (PMC7464147; doi:10.3390/cancers12082005)
Supplement: Supplementary file 1 [file cancers-12-02005-s001.zip › cancers-859052-SI.docx]

Supplementary Material

1. Comparison of Gene Expressions in Patient Sample Cohorts, Correlation Analysis, and Hierarchical Clustering

UCSC Xena, an online exploration tool for public and private, multi-omic, and clinical/phenotype data, was used to evaluate expression of various selected genes across samples [1]. To compare tumor vs. normal samples, TCGA TARGET GTEx study was used and data were filtered down to pancreatic samples (Figure 1, 4). The gene expression dataset normalized by the upper quartile method ('RSEM norm__count' dataset) was utilized for visualization of expression levels of selected genes. Correlation analysis was performed in Instant Clue [2], a software suite for interactive data visualization and analysis, and Pearson correlation coefficient was calculated (Figure 4B).

GSE19650 series from GEO repository (NCBI) in the form of raw fluorescence CEL file was downloaded into Bioinformatics Array Research Tool (BART) [3], a R Shiny web application, parsed, normalized, and log2 transformed. Normalized expression data were further processed using Instant Clue, a software suite for interactive data visualization and analysis, correlation analysis (Pearson correlation coefficient), and hierarchical clustering (euclidean distance, complete linkage) were performed (Figure 7 and 8).

2. Bioinformatics Analysis

In silico analysis of the CA9 promoter was performed using the JASPAR ([http://jaspar.genereg.net)](http://jaspar.genereg.net)/) [4] and the MatInspector ([https://www.genomatix.de)](https://www.genomatix.de)/) [5,6]. The promoter sequence was extracted directly from the ElDorado genome database after the CA9 gene submission. The accurate position of predicted binding elements was calculated according to the transcription start site (TSS).

3. Patient Cohort

Fifty-five patients with a diagnosis of PDAC undergoing surgery with curative intent between 2015 and 2016 at the Department of Surgery, Medical University of Vienna were retrospectively defined as study cohort and the corresponding FFPE tumor samples were prepared for further analysis. Patient and tumor characteristics were collected from the institutional database. The study was approved by the local ethics committee of the Medical University of Vienna (“Ethikkommission”, protocol no. 1753/2014).

4. Immunohistochemical Staining of Resected PDAC Samples

Dissected tissues embedded in paraffin were processed according to the standard histological procedure. Immunohistochemistry was performed as described previously [7]. Briefly, sections were stained using DakoCytomation EnVision^®^ + System-HRP (DAB) with specific anti-CA IX antibody M75 ([8]; hybridoma medium diluted 1:100) for 1 h at room temperature. Staining was visualized with DAB solution with 3, 3'-diaminobenzidine as a chromogenic substrate. Finally, the sections were counterstained with Mayer's hematoxylin and mounted in Aquamount (Merck, Darmstadt, Germany). The stained sections were examined and scanned using 3DHISTECH Pannoramic MIDI Slidescanner and analyzed using Pannoramic Viewer 1.15.4 Software.

References

1. Goldman, M.J.; Craft, B.; Hastie, M.; Repecka, K.; McDade, F.; Kamath, A.; Banerjee, A.; Luo, Y.; Rogers, D.; Brooks, A.N., et al. Visualizing and interpreting cancer genomics data via the Xena platform. *Nat. Biotechnol.* **2020**, *38*, 675-678, doi:10.1038/s41587-020-0546-8.
2. Nolte, H.; MacVicar, T.D.; Tellkamp, F.; Kruger, M. Instant Clue: A Software Suite for Interactive Data Visualization and Analysis. *Sci. rep.* **2018**, *8*, 12648, doi:10.1038/s41598-018-31154-6.
3. Amaral, M.L.; Erikson, G.A.; Shokhirev, M.N. BART: bioinformatics array research tool. *BMC Bioinform.* **2018**, *19*, 296, doi:10.1186/s12859-018-2308-x.
4. Fornes, O.; Castro-Mondragon, J.A.; Khan, A.; van der Lee, R.; Zhang, X.; Richmond, P.A.; Modi, B.P.; Correard, S.; Gheorghe, M.; Baranasic, D., et al. JASPAR 2020: update of the open-access database of transcription factor binding profiles. *Nucleic acids research* **2020**, *48*, D87-D92, doi:10.1093/nar/gkz1001.
5. Cartharius, K.; Frech, K.; Grote, K.; Klocke, B.; Haltmeier, M.; Klingenhoff, A.; Frisch, M.; Bayerlein, M.; Werner, T. MatInspector and beyond: promoter analysis based on transcription factor binding sites. *Bioinformatics* **2005**, *21*, 2933-2942, doi:10.1093/bioinformatics/bti473.
6. Quandt, K.; Frech, K.; Karas, H.; Wingender, E.; Werner, T. MatInd and MatInspector: new fast and versatile tools for detection of consensus matches in nucleotide sequence data. *Nucleic Acids Res.* **1995**, *23*, 4878-4884, doi:10.1093/nar/23.23.4878.
7. Takacova, M.; Bartosova, M.; Skvarkova, L.; Zatovicova, M.; Vidlickova, I.; Csaderova, L.; Barathova, M.; Breza, J., Jr.; Bujdak, P.; Pastorek, J., et al. Carbonic anhydrase IX is a clinically significant tissue and serum biomarker associated with renal cell carcinoma. *Oncol. Lett.* **2013**, *5*, 191-197, doi:10.3892/ol.2012.1001.
8. Pastorekova, S.; Zavadova, Z.; Kostal, M.; Babusikova, O.; Zavada, J. A novel quasi-viral agent, MaTu, is a two-component system. *Virology* **1992**, *187*, 620-626, doi:10.1016/0042-6822(92)90464-z.

| 1. 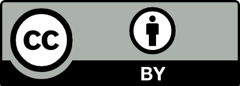 | 1. © 2020 by the authors. Submitted for possible open access publication under the terms and conditions of the Creative Commons Attribution (CC BY) license (http://creativecommons.org/licenses/by/4.0/). |
| --- | --- |
